# A Network Medicine Approach for Drug Repurposing in Duchenne Muscular Dystrophy

**DOI:** 10.3390/genes12040543

**Published:** 2021-04-09

**Authors:** Salvo Danilo Lombardo, Maria Sofia Basile, Rosella Ciurleo, Alessia Bramanti, Antonio Arcidiacono, Katia Mangano, Placido Bramanti, Ferdinando Nicoletti, Paolo Fagone

**Affiliations:** 1 Department of Structural & Computational Biology at the Max Perutz Labs, University of Vienna, 1010 Vienna, Austria; salvo.lombardo.sdl@gmail.com; 2 IRCCS Centro Neurolesi “Bonino-Pulejo”, Via Provinciale Palermo, Contrada Casazza, 98124 Messina, Italy; sofiabasile@hotmail.it (M.S.B.); rossella.ciurleo@irccsme.it (R.C.); alessia.bramanti@gmail.com (A.B.); placido.bramanti@irccsme.it (P.B.); 3 Department of Biomedical and Biotechnological Sciences, University of Catania, Via S. Sofia 89, 95123 Catania, Italy; a.arcidiacono@unict.it (A.A.); kmangano@unict.it (K.M.); paolofagone@yahoo.it (P.F.)

**Keywords:** Duchenne muscular dystrophy, microarray analysis, network medicine, protein–protein interactions, drug discovery, drug repurposing, computational biology

## Abstract

Duchenne muscular dystrophy (DMD) is a progressive hereditary muscular disease caused by a lack of dystrophin, leading to membrane instability, cell damage, and inflammatory response. However, gene-editing alone is not enough to restore the healthy phenotype and additional treatments are required. In the present study, we have first conducted a meta-analysis of three microarray datasets, GSE38417, GSE3307, and GSE6011, to identify the differentially expressed genes (DEGs) between healthy donors and DMD patients. We have then integrated this analysis with the knowledge obtained from DisGeNET and DIAMOnD, a well-known algorithm for drug–gene association discoveries in the human interactome. The data obtained allowed us to identify novel possible target genes and were used to predict potential therapeutical options that could reverse the pathological condition.

## 1. Introduction

The dystrophinopathies are a spectrum of X-linked muscle diseases that include Duchenne muscular dystrophy (DMD), Becker muscular dystrophy (BMD), and DMD-associated dilated cardiomyopathy (DCM) [1].

The lack of functional dystrophin leads to membrane instability that alters the normal muscle development, causing structural and metabolic changes and, consequently, leading to a chronic inflammatory response [2,3]. DMD usually presents in early childhood with delayed motor milestones, including delays in walking independently and standing up from a supine position. However, this clinical feature rapidly evolves, forcing affected children to be wheelchair-dependent by the age of 12 years. The natural course of the disease is characterized by impairment of the cardiac muscle that leads to a dilated cardiomyopathy, with congestive heart failure responsible for premature patient death [4,5]. The standard of care treatment is represented by corticosteroids [6]; however, new lines of research are currently being explored and applied, such as the possibility to restore the function of dystrophin with gene addition, exon skipping [7,8,9], stop codon readthrough [10,11], microdystrophin transfer with adeno-associated virus [12], and genome-editing therapies [13]. However, it is still very important to find new additional treatments that can reduce the symptoms, slow disease progression, and increase overall survival. To achieve this goal, the repurposing of drugs currently used for other clinical indications represents an attractive option in drug discovery, reducing development costs and time [14]. Computational approaches and network analysis have been extensively and successfully applied to find new gene–disease associations [15,16] and to propose new mechanisms of action for known drugs [17,18].

**Figure 1:**
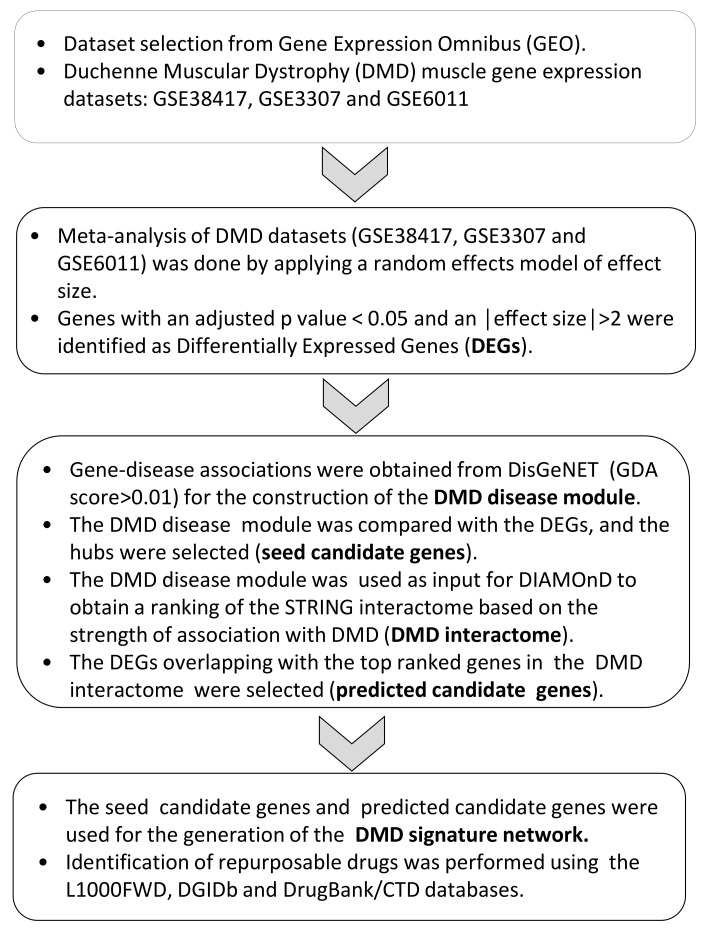
Study layout.

Here, we have first defined the disease module for DMD, by extracting 375 gene–disease associations from DisGeNET [19], and then, by using this module as input for DIAMOnD [20], we were able to discover new gene–disease associations that we integrated with transcriptomic expression obtained by the meta-analysis of three microarray datasets—GSE38417, GSE3307, and GSE6011. After this, we focused our attention on the hubs of this overlap, considering them as possible drug targets. Finally, we explored potential therapeutical options using a transcriptomic anti-signature approach (via L1000FDW [21]) and gene–drug interactions (via DGIdb [22], DrugBank [23], and the Comparative Toxicogenomics Database [24]). A summary pipeline of the study is presented in Figure 1.

## 2. Materials and Methods

### 2.1. Dataset Selection and Analysis

The NCBI Gene Expression Omnibus (GEO) database (http://www.ncbi.nlm.nih.gov/geo/; accessed on 2 February 2021) was used to identify high-throughput gene expression datasets comparing muscle transcriptomic profiles from healthy donors vs. DMD patients. Inclusion criteria for the selection of the datasets were as follows: (i) whole-genome gene expression data; (ii) the datasets should contain both cases and matched controls; (iii) human muscle samples. Three datasets were included in the meta-analysis: GSE38417, GSE3307, and GSE6011 (Table 1). Briefly, the GSE38417 dataset included 6 samples of skeletal muscle biopsy from healthy people and 16 samples from DMD patients. The GSE3307 dataset included 17 samples of skeletal muscle biopsy from healthy people and 10 samples from DMD patients. Biopsies were taken at the time of diagnosis and were from the vastus lateralis muscle [25,26]. Normal muscle biopsies were from healthy donors participating in exercise physiology studies [25,26]. The GSE6011 dataset included 14 samples of controls and 23 samples from DMD patients [27]. None of the patients were or had been under corticosteroid treatment at the time of biopsy. All samples were taken from the quadriceps. DMD patients’ age was 14.9 ± 14.6 months, while control donors were 25.7 ± 29.9 months old [27]. Control samples did not have signs of muscle pathology as determined by histological and histochemical analysis [27].

For the meta-analysis of GSE38417, GSE3307, and GSE6011, a random-effects model of effect size was used to integrate gene expression patterns from the two datasets. Genes with an adjusted *p* value (FDR) < 0.05 and an |effect size| > 2 were identified as differentially expressed genes (DEGs) and selected for further analysis. The random-effects model allows the effect size to vary from study to study by incorporating unknown cross-study heterogeneities in the model. The meta-analysis was performed using the web utility, NetworkAnalyst (https://www.networkanalyst.ca/; accessed on 2 February 2021) [28], which uses the metaMA package [29].

### 2.2. Enrichment Analysis and Sub-Cluster Identification

Functional enrichment analysis was conducted using the web-based utility, Metascape [30]. Metascape analysis is based on publicly available databases, e.g., Kyoto Encyclopedia of Genes and Genomes (KEGG), Gene Ontology, MSigDB (Molecular Signatures Database), and Reactome [30]. Metascape automatically aggregates enriched terms into non-redundant groups by calculating the pairwise similarity between any two terms [30].

Relationships among the enriched terms were visualized as a network in Cytoscape [31], considering an edge between two terms if they showed a similarity score > 0.3. No more than 15 terms per cluster and no more than 250 terms in total were considered.

### 2.3. Network Construction, Seed Gene Identification, and Disease Propagation Algorithms

The STRING [32] database was used to construct the protein–protein interaction network. Interaction data were defined as physical interaction, co-expression, predicted, co-localization, pathway, genetic interactions, and shared protein domains. An interaction score of 0.7 was used as a threshold to build the network (high confidence). The seed genes associated with DMD were selected from DisGeNET [19] (https://www.disgenet.org/; accessed on 2 February 2021), considering a gene disease association (GDA) score > 0.01, in order to obtain a DMD disease module. DisGeNET is one of the largest publicly available databases containing collections of genes and variants associated with human diseases [19]. DisGeNET integrates data from expert-curated repositories, GWAS catalogues, animal models, and the scientific literature. The DMD disease module was compared with the DEGs, and the hubs (defined as nodes with at least 20 interactions in the disease module) were selected (seed candidate genes). The DMD disease module was used as input for DIAMOnD to obtain a ranking of the STRING interactome based on the strength of association with DMD (DMD interactome). DIAMOnD [20] is an algorithm used to propagate the disease module in the network and find new gene–disease associations, computing a particular quantity, defined as connectivity significance, which provides a ranking of all the genes in the network based on the strength of association with the seed genes. The DEGs overlapping with the highest-ranked genes in the DMD interactome were selected (predicted candidate genes). The seed candidate genes and the predicted candidate genes were used for the generation of the DMD signature network. A layout of the experimental design is presented in Figure 1.

### 2.4. Drug Identification

For the prediction of drugs potentially repurposable for use in DMD, three different approaches were used in the present study.

First, the L1000FDW web-based utility was used to identify potential drugs for the treatment of DMD, based on the anti-signature perturbation analysis [21]. L1000FWD computes the anti-similarity between an input gene expression profile and data from the Library of Integrated Network-based Cellular Signatures (LINCS)-L1000, in order to prioritize drugs potentially able to reverse the input transcriptional feature [21]. The genes belonging to the DMD signature network were used as input genes.

Secondly, the last updated version of the gene–drug interaction dataset from DGIdb [22] (https://www.dgidb.org/; accessed on 2 February 2021) was downloaded and queried (Nov 2020) in order to select the drugs that can modulate the DMD signature genes. DGIDb is a comprehensive, free-to-access, online database containing information on drugs and drug targets and their interactions. The whole database was divided in two different sub-tables, collecting the drugs that have an upregulated action and the ones that instead downregulate, respecting the following filtering. Drugs with one of the following types of interactions were considered with a downregulated action: “agonist, inhibitor”, “allosteric modulator, antagonist”, “antagonist”, “antagonist, activator”, “antagonist, agonist”, “antagonist, allosteric modulator”, “antagonist, antibody”, “antagonist, binder”, “antagonist, blocker”, “antagonist, inducer”, “antagonist, inhibitor”, “antagonist, inverse agonist”, “antagonist, ligand”, “antagonist, ligand, partial agonist”, “antagonist, multitarget”, “antagonist, partial agonist”, “antagonist, potentiator”, “antagonist, substrate”, “antibody, inhibitor”, “binder, inhibitor”, “blocker”, “blocker, inhibitor”, “cleavage”, “inhibitor”, “inhibitor, activator”, “inhibitor, antibody”, “inhibitor, inducer”, “inhibitor, substrate”, “inhibitory allosteric modulator”, “inhibitory allosteric modulator, antagonist”, “inverse agonist”, “ligand, inhibitor”, “modulator, antagonist”, “modulator, inhibitor”, “negative modulator”, “negative modulator, agonist”, “negative modulator, agonist, antagonist”, “negative modulator, agonist, inhibitor”, “negative modulator, antagonist”, “negative modulator, binder, inhibitor”, “negative modulator, inhibitor”, “partial antagonist”, “suppressor”. Drugs that were classified with the following mode of action were considered as activating drugs: “activator”, “activator, inducer”, “adduct”, “agonist”, “agonist, activator”, “agonist, allosteric modulator”, “agonist, inducer”, “agonist, positive modulator”, “agonist, potentiator”, “agonist, stimulator”, “binder, activator”, “inducer”, “inducer, substrate”, “ligand, inducer”, “modulator, activator”, “modulator, inducer”, “modulator, ligand”, “positive modulator”, “potentiator”, “potentiator, activator”, “potentiator, binder”, “stimulator”.

These data sources were used to select the drugs that can modulate our gene candidates in DMD.

Finally, a network-based approach for drug identification was applied to the DMD signature network. A drug–gene network was first constructed by mapping the DMD signature genes to the molecular interaction databases, DrugBank (Version 5.0) and Comparative Toxigenomics Database (CTD) (November 2016). The drugs were then ranked on the basis of their degree of distribution in the drug–gene network and the top 50 drugs were selected for further analysis.

Only drugs and molecules already approved for clinical use or registered in clinical trials were included in all the analyses.

### 2.5. Statistical Analysis

Drugs from the DGIDb database were selected based on a hypergeometric test assessing the significance of the overlap between the DMD signature and the total number of target genes of each drug, considering as background the number of genes in common between the meta-analysis and the STRING database (*n* = 10,575). An adjusted *p* value (FDR) < 0.05 was considered for statistical significance.

For the network-based prediction of the drugs, we first computed for each drug the expected mean and standard deviation of the degree of distribution by generating random gene lists of the same size of the input list. Then, we computed a z-score for deviation from the expected value, and the significance was calculated. An adjusted *p* value (FDR) < 0.05 was considered for statistical significance.

## 3. Results

### 3.1. Meta-Analysis of Gene Expression in DMD

A total of 37 samples from healthy controls and 49 skeletal muscle biopsies from DMD patients were used in the meta-analysis. The meta-analysis identified 735 DEGs between healthy and DMD samples, among which 234 were downregulated and 501 were upregulated in DMD (the complete list of DEGs is available in Table S1).

### 3.2. DMD Module Identification and New Associated Gene Prediction

Genes associated with DMD were selected from DisGeNET, considering a GDA score > 0.01. We found 375 genes associated with DMD, which were used as seed genes (input) for DIAMOnD. These 375 genes were used to create the disease module for DMD. Its largest connected component is represented by 260 nodes and 1101 interactions (Supplementary Figure S1). When compared to a 10.000 subnetwork of the same size, the largest connected component for DMD was much higher compared to the expectation (z-score: 8.9), proving its higher clustering compared to random sampling and validating the common biological role of the disease module (Supplementary Figure S2).

### 3.3. Candidate Gene Identification

**Figure 2:**
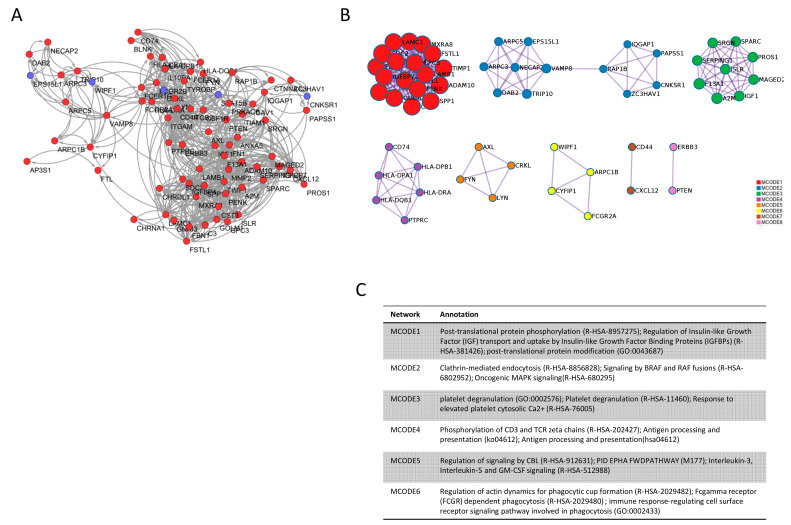
DMD signature network and MCODE analysis. (**A**) Network of the seed and predicted candidate genes (77 genes). Red nodes are the upregulated genes in the DMD signature, while blue nodes are the downregulated genes in the DMD signature; (**B**) MCODE analysis of the genes in the DMD signature network (seed and predicted candidate genes); (**C**) Gene ontology annotation for the MCODE clusters identified in the DMD signature network. The top three significant terms are indicated.

Using the DMD disease module network identified from DisGeNET as input, DIAMOnD was applied to obtain a ranking of the whole STRING interactome based on the strength of association with the respective disease. We then computed the overlap between the DEGs from the meta-analysis and the DMD disease module, obtaining 46 genes. Among these 46 genes, we found five hubs (SPP1, IGF1, FN1, TIMP1, MMP2) in the DMD disease module network, defined as seed candidate genes. Additionally, the DEGs in common with the first 735 predicted genes from DIAMOnD were considered as predicted candidate genes and included 75 genes, among which 72 were hubs in the general STRING interactome. Among the predicted candidate genes, only five genes were downregulated (STAT5B, CRKL, TRIP10, EPS15L1, CNKSR1) and 67 upregulated in DMD. Overall, the seed candidate genes and the predicted candidate genes defined the DMD signature network (Figure 2A). The comprehensive list of the genes forming the DMD signature network is presented in Table S2.

MCODE clustering analysis of the DMD signature network revealed significant enrichment for the terms related to post-translational modifications (MCODE1), clathrin-mediated endocytosis (MCODE2), platelet degranulation (MCODE3), ubiquitination and proteasomal degradation (MCODE5), and immune response (MCODE4 and 6) (Figure 2B,C).

### 3.4. Drugs Associated with DMD

Three different methodologies were applied for the prediction of potential repurposable drugs for DMD. First, the up- and downregulated genes from the predicted candidate genes and the seed candidate genes were used to perform the anti-signature perturbation analysis. We enlisted the potential drugs identified by the L1000FWD analysis in Table 2. Among them, the top drugs with clinical application were emetine and homoharringtonine, followed by prednisolone and testosterone (Table 2).

Next, the DGIDb database was used for drug prediction, by identifying drugs able to modulate the genes belonging to the DMD signature. When considering the drugs that could have a specific action to reverse the DMD phenotype, 27 drugs were found to reach statistical significance (Table 3). Most of these drugs are used or under study for their use in oncology, mainly in hematological cancers. They share many of their targets, so that, in total, only nine genes are effectively drug targets (ITGB2, ITGAM, LYN, CSF1R, FYN, AXL, CD74, ERBB3, A2M). The top three drugs were Rovelizumab, Dasatinib, and Ilorasertib (these last two share the same gene targets).

**Figure 3:**
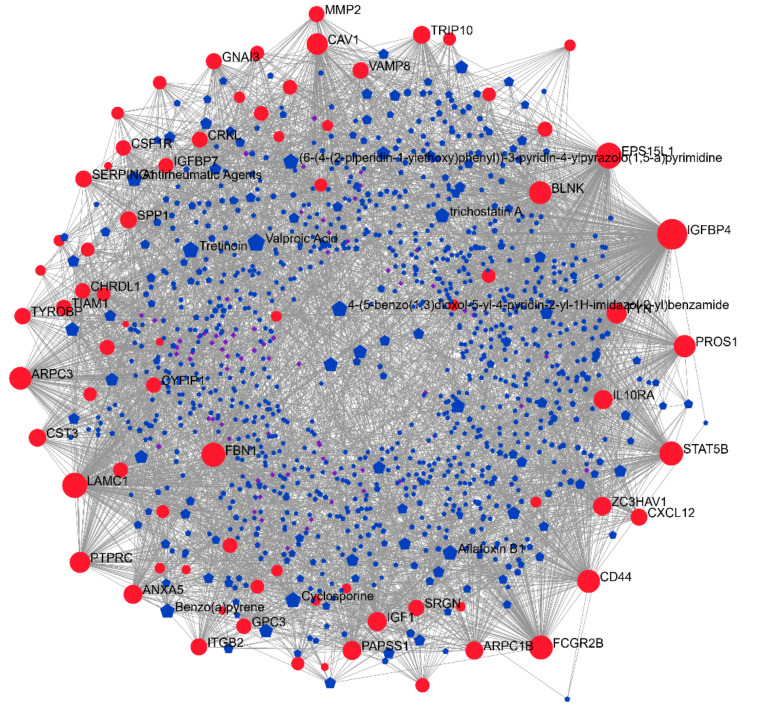
Network analysis of the drug–gene interactome. A network-based approach, based on the DrugBank and CTD databases, was applied for the prediction of drugs potentially repurposable for DMD. Red nodes represent genes belonging to the DMD signature, blue nodes represent drugs from the DrugBank database, and violet nodes represent chemicals from the CTD database.

Finally, a network-based approach, using the DrugBank and CTD databases, was applied (Figure 3). The complete list of the identified drugs is presented in Table 4. The top three drugs were: Cytarabine, an antimetabolite; entinostat, a synthetic benzamide derivative histone deacetylase (HDAC) inhibitor; and isotretinoin, a stereoisomer of all-trans retinoic acid (Table 4). Interestingly, besides entinostat, other HDAC inhibitors were identified, i.e., trichostatin A, vorinostat, and panobinostat (also found in the L1000FDW analysis). Additionally, testosterone was found in both the L1000FDW-based and in the DrugBank/CTD-based analyses (Table 2 and Table 4). Moreover, corticosteroids were commonly identified by L1000FDW (prednisolone and hydrocortisone) and in the network-based analysis (dexamethasone) (Table 2 and Table 4).

## 4. Discussion

DMD is the most common dystrophy in children, with a worldwide prevalence of approximately 0.5 cases per 10,000 male births [33]. Current treatments manage only to alleviate the symptoms and are based on a multidisciplinary approach. For this reason, new therapeutical options are required. Here, we use computational techniques to predict new potential drugs to be tested and validated in the future.

We defined a DMD disease module, consisting of 375 genes, which of 326 appear in the STRING network. We then found that the largest connected component of this module (260 genes) was bigger compared to a random expectation of a 10.000 gene set of the same size (z-score: 8.9) (Supplementary Figure S1). This finding validates the common biological role of the selected genes and so also the choice of our disease module. We then compared the DMD module with the 735 DEGs from the meta-analysis, obtaining an overlap of 46 genes, of which five were hubs in the sub-network (more than 20 interactors) and all of them were upregulated in the meta-analysis (SPP1, IGF1, FN1, TIMP1, and MMP2). We defined these five genes as seed candidate genes and further analyzed them to find new drugs that can invert their expression.

We used the DMD disease module as input for DIAMOnD. This algorithm is very useful to find new gene–disease associations and it has largely proven its efficacy in other diseases [34,35,36]. However, DIAMOnD alone has the limitation of basing its ranking on the connectivity properties of the network, ignoring additional biological information. For this reason, we overlapped the 735 DEGs with the 735 highest-ranked genes from DIAMOnD, integrating the transcriptional expression into our predictions. We found 75 genes in common, of which 72 were hubs in the STRING interactome, which we defined as predicted candidate genes. Among these 72 genes, only five were downregulated (STAT5B, CRKL, TRIP10, EPS15L1, and CNKSR1). Seed and predicted candidate genes formed together the DMD signature network (Figure 2B).

The gene ontology enrichment analysis of the DMD signature network showed that a major role in the development of the disease is played by the immune responses and immunological activation (Figure 2C). This is in agreement with our previous findings, showing the involvement of the proinflammatory cytokine Macrophage Migration Inhibitory Factor (MIF) in DMD pathogenesis [37]. In accordance with our previous data, in this study, we found that two receptors for MIF (CD74 and CD44) are hubs in the DMD signature network and could hence represent potential drug targets. Indeed, milatuzumab, a monoclonal antibody directed against CD74, was predicted by our analysis.

We provide here the first attempt to integrate different data sources and methods to predict new drugs that can be helpful for DMD. By employing different approaches, we cover different biological aspects that take into account transcriptomic expression (via L1000FWD) and pharmacokinetics and pharmacodynamics information, via DGIDb and DrugBank/CTD.

We found new possible drugs useful to reverse the transcriptomic changes of the genes in the DMD signature network using the L1000FWD (Table 3). Emetine and homoharringtonine are the top two drugs that have been predicted to be able to modulate these pathological genes. It should be noted that homoharringtonine has never been previously associated with DMD and muscular dystrophies in general. On the other hand, it is surprising that we observed that emetine was the top predicted drug in the L1000FDW analysis, as it was previously associated with myopathy, both in rats [38] and in humans [39]. However, we may speculate that emetine could exert beneficial effects in DMD as it strongly induces PGC-1α [40], and transgenic expression of PGC-1α in skeletal muscle has been indeed shown to ameliorate muscle damage and to improve locomotive function in mdx mice, a model for DMD [41].

Considering the top drugs able to modulate the predicted candidate genes, both Dasatinib and Ilorasertib share the same target genes (LYN, CSF1R, FYN) and are used as anti-cancer drugs. On the other hand, Rovelizumab is a humanized monoclonal antibody studied for hemorragic shock, stroke, and multiple sclerosis. Rovelizumab has not been previously associated with DMD; however, we show that it is able to modulate two predicted candidate genes (*ITGB2, ITGAM*) that are associated with DMD in mdx mice [42,43].

Interestingly, Dasatinib has already been previously studied as a potential treatment for DMD [44], decreasing the levels of β-dystroglycan phosphorylation on tyrosine and increasing performance on dystrophies animal models (zebrafish and mice) [44,45]. Most of the drugs predicted using the DGIDb database are used in hematological cancers and disorders, in line with the findings of the MCODE enrichment analysis, showing the crucial role of platelet degranulation and Interleukin-3, Interleukin-5, and GM-CSF signaling.

We also interrogated the DrugBank and the CTD databases in order to predict drugs that can modulate the genes in the DMD signature by performing a network analysis (Figure 3). Interestingly, three HDAC inhibitors were predicted, namely entinostat, trichostatin A, and Panobinostat. This is in line with a previous report from Bajanca and Vandel, who showed that trichostatin A prevents early muscle damage in a zebrafish model of DMD [46].

Among the predicted drugs, we also found valproic acid, which is reported to have a particularly increased muscular tropism acting as a negative modulator for GSK3b and ABCB1 and as a positive regulator of CASP8 and ESR2. This could be a good resource for DMD, since ESR2 induces muscular differentiation in facioscapulohumeral muscular dystrophy [47] and GSK3b could help to reduce muscular degeneration [48,49]. Furthermore, due to its neurotropism, valproic acid was also reported to improve neural damage in DMD [50].

In line with the immunological activity in DMD, corticosteroids are currently used for DMD [6] and, along the same line, also other immunomodulators have been recently tested for their possible efficacy. For instance, Cyclosporine A was previously suggested and tested for its potential ability to increase muscular force in DMD. However, contrasting results have been obtained [51,52]. Here, we identified Cyclosporine A as a drug candidate from DrugBank, and corticosteroids were found among the top predicted drugs—more specifically, prednisolone and hydrocortisone in the L1000FDW analysis, and dexamethasone in the network-based analysis.

Moreover, both our approaches (L1000FDW-based and the DrugBank/CTD-based analyses) have identified testosterone as a beneficial treatment in DMD (Table 2 and Table 4). Testosterone administration was recently tested in a small cohort study of 15 adolescents affected by DMD, giving positive results [53].

The unsupervised prediction of drugs currently used for the treatment of DMD is worth mentioning, as it underlines the validity of our methodological approach and of the obtained results.

We found a significant (*p* < 0.001) overlap (49.3%) between the genes in the DMD signature network and the genes altered in BMD (Supplementary Figure S3). For this reason, we believe that these findings can be helpful not only for DMD but also for other similar diseases, such as BMD, where there is a common genetic origin.

This study represents the first attempt to integrate different data sources and approaches to find new, efficient drugs for DMD. Due to the high number of existing drugs, computational approaches are very helpful to test millions of possible associations and perform a pre-screening to be validated then in biological experiments [14]. In the past, network approaches alone have been used to reach the goal of drug repurposing [18]. There are some limitations in our study; for example, a better annotation of the metadata of the biological samples would help to cluster together similar biological patterns, reducing the heterogeneous complexity (adjustment for different types of mutations and for previous treatments). We are also aware that further biological knowledge should be considered and biological validation is necessary to prove our findings. However, we believe that our approach is a good proof of concept to combine different biological types of information and lead to new insights to be then validated in vivo.

## 5. Conclusions

In the last decade, new therapeutic perspectives have been explored and used for dystrophinopathies. Although improvements in patient treatment and management have been able to slow down disease progression [13], DMD still represents an unmet medical need. For this reason, new types of treatments are required. Computational techniques and big data analysis are very helpful to reach the aim of drug discovery and repurposing. In this work, we have explored existing gene–disease associations and predicted new ones, combining techniques of network analysis and transcriptomic expression to identify possible drug targets. To accomplish this aim, we have integrated many different databases and techniques, taking into account drug–gene relationships and transcriptomic changes (L1000FWD, DGIdb, DrugBank, and CTD). From our findings, we have identified similarities with hematological diseases and highlight 77 possible gene targets, among which nine (ITGB2, ITGAM, LYN, CSF1R, FYN, AXL, CD74, ERBB3, A2M) are already druggable. We present here the first attempt to create a comprehensive overview of the possible drugs that can be beneficial for DMD and potentially for other diseases with the same genetic background, such as BMD.

## Figures and Tables

**Table 1:** Datasets included in the meta-analysis.

Accession Number	Tissue	Samples	Platform
GSE38417	Muscle	16 DMD patients and 6 healthy controls	Affymetrix Human Genome U133 Plus 2.0
GSE3307	Muscle	10 DMD patients and 17 healthy controls	Affymetrix Human Genome U133A and U133B
GSE6011	Muscle	23 DMD patients and 14 healthy controls	Affymetrix Human Genome U133A

**Table 2:** Drugs predicted by the L1000FDW software.

Sig_ID	Drug	Drug Category	Indication	Similarity Score	*p*-Value	*q*-Value	Z-Score	Combined Score
CPC007_A375_24H:BRD-K03067624-003-19-3:10	emetine	emetine alkaloids	anti-protozoal	−0.1948	1.40 × 10^−11^	2.85 × 10^−8^	1.78	−19.35
CPC017_A375_6H:BRD-K76674262-001-01-7:10	homoharringtonine	cephalotaxus alkaloids	CML	−0.1818	1.32 × 10^−10^	1.49 × 10^−7^	1.71	−16.86
CPC004_VCAP_24H:BRD-A01643550-001-03-1:10	prednisolone	synthetic glucocorticoid	anti-inflammatory or immunosuppressive agent	−0.1688	2.09 × 10^−9^	1.01 × 10^−6^	1.81	−15.74
CPC002_VCAP_24H:BRD-K90553655-001-03-6:10	testosterone	anabolic steroid	hypogonadism	−0.1688	2.42 × 10^−9^	1.12 × 10^−6^	1.83	−15.77
CPC017_HT29_6H:BRD-K07691486-001-04-9:10	roscovitine	synthetic organic	kinase inhibitor	−0.1558	1.58 × 10^−8^	4.10 × 10^−6^	1.67	−13.04
CPD002_PC3_24H:BRD-K08547377-394-01-9:10	irinotecan	DNA replication inhibitor	colon cancer	−0.1429	1.39 × 10^−7^	2.06 × 10^−5^	1.66	−11.38
CPC006_PC3_6H:BRD-K82135108-001-01-9:10	elesclomol	sulfur compounds	anti-cancer activity	−0.1429	1.36 × 10^−7^	2.06 × 10^−5^	1.77	−12.16
PCLB002_A375_24H:BRD-K02130563:1.11	panobinostat	non-selective histone deacetylase (HDAC) inhibitor	multiple myeloma and other cancers	−0.1429	2.04 × 10^−7^	2.55 × 10^−5^	1.59	−10.64
MUC.CP006_MCF7_6H:BRD-K77987382-001-08-2:10	mebendazole	benzimidazole	antihelmintic	−0.1429	1.22 × 10^−7^	1.97 × 10^−5^	1.63	−11.26
CPC005_PC3_24H:BRD-A07000685-001-03-6:10	hydrocortisone	adrenal glucocorticoid	immune and allergic disorders, adrenal insufficiency disorders	−0.1429	1.92 × 10^−7^	2.45 × 10^−5^	1.78	−11.93

**Table 3:** Drugs predicted using the DGIDb database.

Drugs	Indication	Number of Target Genes among the Predicted Candidate Genes	Predicted Candidate Genes	Number of Total Target Genes	*p*-Value	FDR
ROVELIZUMAB	Hemorrhagic shock, multiple sclerosis, stroke	2	*ITGB2, ITGAM*	3	1.37 × 10^−4^	0.012558
DASATINIB	Chronic myelogenous leukemia, acute lymphoblastic leukemia	3	*LYN, CSF1R, FYN*	23	4.86 × 10^−4^	0.014904
ILORASERTIB	Phase II Study for CDKN2A deficient solid tumors	3	*LYN, CSF1R, FYN*	21	3.69 × 10^−4^	0.014904
NINTEDANIB	Idiopathic Pulmonary Fibrosis, NSCLC	2	*LYN, FYN*	19	0.007	0.029273
JNJ-26483327	Phase I study for solid tumors	2	*LYN, FYN*	9	0.002	0.029273
ACALABRUTINIB	Mantle cell lymphoma	2	*LYN, FYN*	14	0.004	0.029273
IBRUTINIB	B cell cancers	2	*LYN, FYN*	16	0.005	0.029273
XL-228	Phase I study for chronic myeloid leukemia	2	*LYN, FYN*	16	0.005	0.029273
PEXMETINIB	Hematological cancers	2	*LYN, FYN*	12	0.003	0.029273
TG100-801	Diabetic macular edema and proliferative diabetic retinopathy	2	*LYN, FYN*	16	0.005	0.029273
ENMD-981693	Phase II study for solid cancers	2	*LYN, FYN*	19	0.007	0.029273
GALLAMINE	Non-depolarizing muscle relaxant	1	*CHRNA1*	1	0.007	0.029273
AME-133V	Phase III study for follicular lymphoma and Phase I for rheumatoid arthritis	1	*ITGB2*	1	0.007	0.029273
ERLIZUMAB	Heart attack, stroke, and traumatic shock	1	*ITGB2*	1	0.007	0.029273
BEMCENTINIB	Phase II study for solid and hematological tumors	1	*AXL*	1	0.007	0.029273
MILATUZUMAB	Multiple myeloma and other hematological tumors	1	*CD74*	1	0.007	0.029273
ARRY-382	Phase II study for advanced solid tumors	1	*CSF1R*	1	0.007	0.029273
MM-121	Phase II study for lung and breast cancer	1	*ERBB3*	1	0.007	0.029273
PATRITUMAB	Phase II study for squamous cell cancer of the head and neck	1	*ERBB3*	1	0.007	0.029273
BOSUTINIB	Chronic myelogenous leukemia	2	*LYN, FYN*	23	0.011	0.044
BAFETINIB	Phase II study for chronic myelogenous leukemia	1	*LYN*	2	0.014	0.046
BACITRACIN	Polypeptide antibiotic	1	*A2M*	2	0.014	0.046
EFALIZUMAB	psoriasis	1	*ITGB2*	2	0.014	0.046
LIFITEGRAST	Keratoconjunctivitis sicca	1	*ITGB2*	2	0.014	0.046
BPI-9016	Phase I study for NSCLC	1	*AXL*	2	0.014	0.046

**Table 4:** Predicted drugs using the DrugBank and the CTD databases.

Rank	Drug	Degree	FDR
1	Cytarabine	12	0
2	Entinostat	32	0
3	Isotretinoin	21	0
4	Paclitaxel	15	0
5	Tretinoin	48	0
6	Mitoxantrone	8	8.45 × 10^−9^
7	Raloxifene Hydrochloride	14	2.54 × 10^−8^
8	Doxorubicin	15	4.6 × 10^−8^
9	Indomethacin	8	2.15 × 10^−7^
10	Simvastatin	8	2.7 × 10^−7^
11	Decitabine	20	1.01 × 10^−6^
12	Curcumin	12	1.53 × 10^−6^
13	Cisplatin	24	2.53 × 10^−6^
14	Dexamethasone	15	2.96 × 10^−6^
15	Ethinyl Estradiol	10	3.51 × 10^−6^
16	Azacitidine	9	4.2 × 10^−6^
17	Trichostatin A	37	4.82 × 10^−6^
18	Aspirin	10	6.71 × 10^−6^
19	Vorinostat	25	9.95 × 10^−6^
20	Ascorbic Acid	8	1.25 × 10^−5^
21	Zoledronic acid	19	1.25 × 10^−5^
22	Resveratrol	24	2.22 × 10^−5^
23	Sulforafan	13	2.3 × 10^−5^
24	Tamoxifen	12	2.82 × 10^−5^
25	Tamibarotene	19	4.06 × 10^−5^
26	Carmustine	8	5.77 × 10^−5^
27	Vitamin E	17	5.77 × 10^−5^
28	Cyclosporine	44	0.000133
29	Etoposide	9	0.000134
30	Carbamazepine	20	0.000136
31	Methotrexate	22	0.000136
32	Panobinostat	14	0.000136
33	Phenobarbital	14	0.000136
34	Calcitriol	21	0.000243
35	Valproic Acid	60	0.000383
36	Genistein	14	0.000496
37	Testosterone enanthate	9	0.00079
38	Fulvestrant	7	0.0013
39	Progesterone	18	0.0013
40	Acetylcysteine	8	0.002457
41	Catechin	8	0.002534
42	Rosiglitazone	9	0.00257
43	Estradiol	30	0.003208
44	Quercetin	20	0.006024
45	Afimoxifene	8	0.013992
46	Troglitazone	8	0.034244
47	Vincristine	8	0.037712
48	Bortezomib	7	0.037951
49	Fluorouracil	12	0.040236

## Data Availability

Gene expression data can be found on the Gene Expression Omnibus (GEO) database (https://www.ncbi.nlm.nih.gov/gds; accessed on 2 February 2021), under accession numbers GSE38417, GSE3307, and GSE6011.

## Supplementary Materials

The following are available online at https://www.mdpi.com/article/10.3390/genes12040543/s1, Figure S1: DMD module network, Figure S2: DMD module network is more connected than a random module of the same size, Figure S3: Shared genes between BMDand DMD signature, Table S1: List of differentially expressed genes in DMD as determined by the meta-analysis of the GSE38417, GSE3307, and GSE6011 datasets, Table S2: List of genes belonging to the DMD signature network.
