# Supplementary material for: A Network Medicine Approach for Drug Repurposing in Duchenne Muscular Dystrophy
Source: Genes (Basel). 2021 Apr 9;12(4):543. doi: 10.3390/genes12040543 (PMC8069953; doi:10.3390/genes12040543)
Supplement: Supplementary file 1 [file genes-12-00543-s001.pdf]

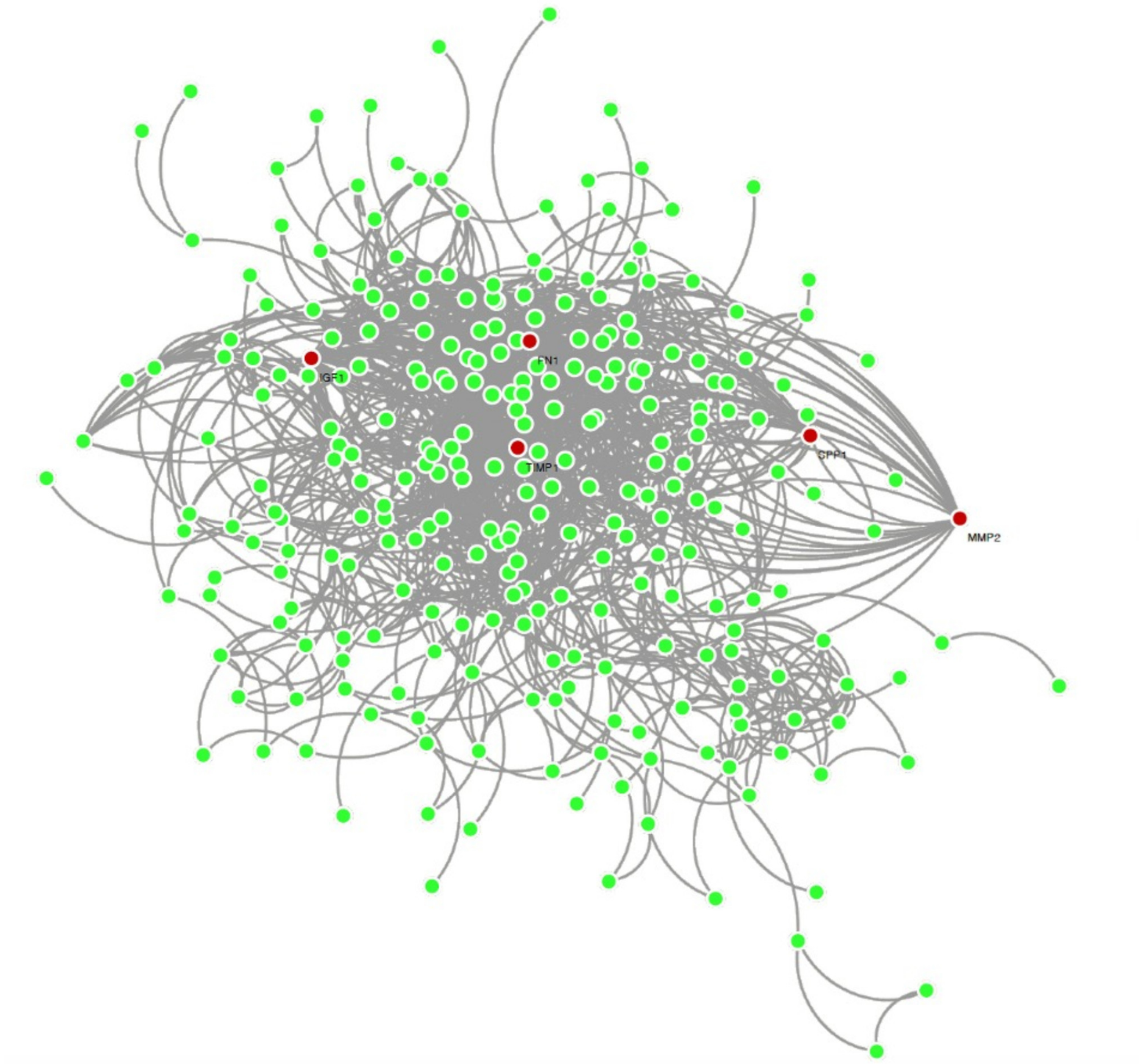

Figure S1. DMD module network. The network is formed by 260 genes from DisGeNET and 1101 interactions from STRING. Red nodes are the five seed candidate genes.

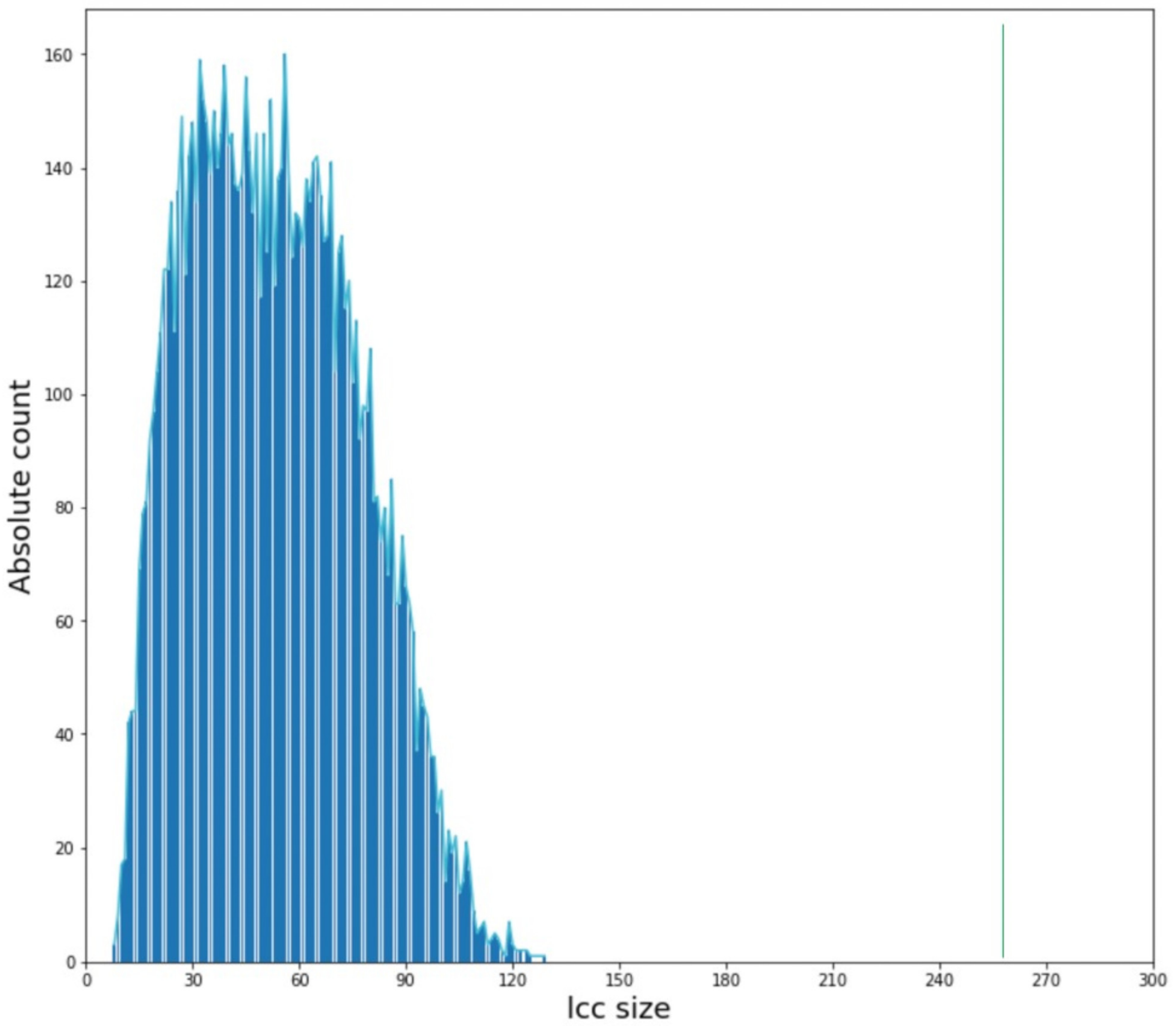

Figure S2. DMD module network is more connected than a random module of the same size. It is shown the distribution of the largest connected component of 10.000 random modules of the same size of the DMD module network. The green line (x=260) represents the DMD largest connected component, obtaining a z-score=8.9.

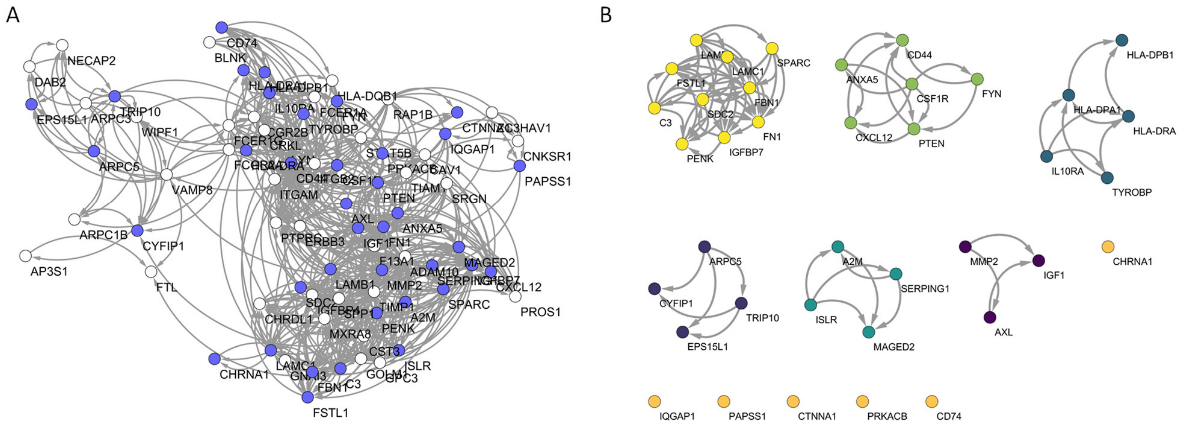

Figure S3. Shared genes between BMD and DMD signature. A) A meta-analysis of three microarray datasets (GSE3307, GSE13608 and GSE109178) was performed for the identification of differentially expressed genes (DEGs) in BMD muscle biopsies as compared to normal muscle biopsies. Briefly, the GSE13608 dataset included 6 samples of skeletal muscle biopsy from healthy people and 5 samples from BMD patients. Biopsies were taken from either biceps brachii, triceps brachii or deltoid. The GSE3307 dataset included 17 samples of skeletal muscle biopsy from healthy people and 10 samples from BMD patients. The GSE109178 dataset included 14 samples of controls and 11 samples from BMD patients. For both GSE3307 and GSE10917 datasets, biopsies were taken at the time of diagnosis and from the vastus lateralis. For the meta-analysis of GSE13608, GSE3307 and GSE109178, a random effects model of effect size measure was used to integrate gene expression patterns from the two datasets. Genes with an adjusted p value (FDR) < 0.05 and an $|\text{effect size}| > 2$ were identified as DEGs and selected for further analysis. A significant number of DEGs ($p<0.001$) were in common with the DMD signature genes (blue nodes), as determined by a hypergeometric test assessing the significance of the overlap between the BMD DEGs and the number of DMD signature genes B) MCODE analysis of the overlapping genes between BMD DEGs and DMD signature genes.

Suppl. Table 1. List of differentially expressed genes in DMD as determined by the meta-analysis of the GSE38417, GSE3307 and GSE6011 datasets.

EntrezID	Name	CombinedES	Pval
1072	CFL1	2.638	0
6282	S100A11	3.1412	0
975	CD81	2.9806	0
6281	S100A10	3.4436	0
3115	HLA-DPB1	2.8816	0
302	ANXA2	4.2067	0
7805	LAPTM5	3.6247	0
8673	VAMP8	2.9953	0
713	C1QB	2.6594	0
6275	S100A4	3.8619	0
2014	EMP3	3.0342	0
1756	DMD	-2.9074	0
4129	MAOB	-3.2433	0
23166	STAB1	3.2344	0
6123	RPL3L	-2.6272	0
5207	PFKFB1	-2.8532	0
10916	MAGED2	3.0779	0
716	C1S	3.3305	0
483	ATP1B3	3.1909	0
3958	LGALS3	2.9697	0
84617	TUBB6	4.0407	0
84790	TUBA1C	3.1103	0
5478	PPIA	3.6242	0
3113	HLA-DPA1	2.9842	0
2512	FTL	3.3021	0
26986	PABPC1	3.024	0
8635	RNASET2	3.5856	0
712	C1QA	3.6703	0

10763	NES	3.4116	0
28959	TMEM176B	3.5987	0
2896	GRN	2.5825	8.1567E-14
2806	GOT2	-2.5331	8.1567E-14
5255	PHKA1	-2.5052	1.4501E-13
7351	UCP2	2.9441	1.4501E-13
4125	MAN2B1	2.5018	1.4501E-13
23175	LPIN1	-2.4784	1.4501E-13
26509	MYOF	3.3517	2.0606E-13
2	A2M	2.4783	2.0606E-13
301	ANXA1	2.4327	2.6771E-13
754	PTTG1IP	2.4189	4.5677E-13
929	CD14	3.3914	4.9717E-13
4069	LYZ	2.414	4.9717E-13
4635	MYL4	2.3894	6.0701E-13
3108	HLA-DMA	3.0577	7.1185E-13
11167	FSTL1	2.3913	7.5404E-13
3689	ITGB2	2.3645	8.3302E-13
5837	PYGM	-2.3712	8.3302E-13
2350	FOLR2	2.3634	9.0556E-13
4191	MDH2	-2.3547	9.0556E-13
53405	CLIC5	-2.3036	1.9837E-12
5236	PGM1	-2.2607	3.6337E-12
5176	SERPINF1	2.6884	3.7646E-12
102	ADAM10	2.2241	6.5992E-12
3728	JUP	-2.2153	7.5404E-12
103	ADAR	2.2042	7.9253E-12
6261	RYR1	-2.2073	8.2965E-12
307	ANXA4	2.2084	1.0441E-11
38	ACAT1	-2.1693	1.7191E-11
558	AXL	2.1538	1.92E-11

7416	VDAC1	-2.9861	2.1273E-11
10579	TACC2	-2.6729	2.3491E-11
633	BGN	2.1371	2.4207E-11
3119	HLA-DQB1	2.1338	2.7676E-11
83442	SH3BGRL3	2.1541	3.226E-11
826	CAPNS1	2.1134	4.0734E-11
1508	CTSB	2.0997	4.0734E-11
9927	MFN2	-2.2507	4.8969E-11
56941	HMCES	-2.0821	5.1665E-11
55037	PTCD3	-2.0709	7.0322E-11
5164	PDK2	-2.0463	8.5724E-11
3055	HCK	2.0552	8.9039E-11
5144	PDE4D	-2.0433	1.0709E-10
4684	NCAM1	2.0401	1.0818E-10
83700	JAM3	2.9123	1.0818E-10
5552	SRGN	2.0239	1.1624E-10
1975	EIF4B	-2.0233	1.2872E-10
1725	DHPS	-2.0175	1.3976E-10
11031	RAB31	3.7843	1.5186E-10
10133	OPTN	-2.5662	1.8355E-10
1462	VCAN	3.4871	2.1581E-10
4478	MSN	2.1058	3.8994E-10
23171	GPD1L	-2.9455	4.144E-10
140461	ASB8	-2.1172	0.000000001
9961	MVP	2.6771	0.000000001
1436	CSF1R	3.5282	1.061E-09
10475	TRIM38	2.6186	1.1889E-09
715	C1R	4.1449	1.3153E-09
51303	FKBP11	3.2183	1.8451E-09
4608	MYBPH	2.7266	1.8637E-09
60	ACTB	2.804	4.418E-09

972	CD74	3.1564	5.6554E-09
10581	IFITM2	2.7676	1.1143E-08
25900	IFFO1	2.4344	1.4398E-08
10410	IFITM3	2.7059	1.5056E-08
103910	MYL12B	2.1665	1.6471E-08
7162	TPBG	2.7137	1.7452E-08
10409	BASP1	2.3413	1.9871E-08
7305	TYROBP	4.0016	2.3284E-08
967	CD63	3.2678	2.6389E-08
3122	HLA-DRA	3.9545	3.1334E-08
3487	IGFBP4	2.0655	4.4073E-08
9093	DNAJA3	-2.3413	4.9241E-08
203068	TUBB	2.8053	0.000000062
150	ADRA2A	2.3073	6.637E-08
1009	CDH11	2.2569	0.000000068
477	ATP1A2	-2.3278	9.8512E-08
4256	MGP	2.9497	1.0728E-07
8407	TAGLN2	2.2887	1.0789E-07
2006	ELN	2.4596	1.1676E-07
9322	TRIP10	-2.8064	1.3088E-07
818	CAMK2G	-2.3523	1.8483E-07
23643	LY96	3.5457	0.000000208
29995	LMCD1	-2.0013	2.3707E-07
10577	NPC2	3.8126	3.205E-07
4689	NCF4	2.3732	3.2277E-07
5912	RAP2B	3.1832	3.2683E-07
2202	EFEMP1	2.3358	3.624E-07
4267	CD99	3.8114	3.8185E-07
5886	RAD23A	-3.6719	3.8513E-07
54587	MXRA8	2.2948	4.8183E-07
2987	GUK1	2.0042	5.0311E-07

5538	PPT1	2.8963	0.000000641
7431	VIM	3.1855	6.9689E-07
6284	S100A13	2.2316	7.3366E-07
5502	PPP1R1A	-2.2662	8.3343E-07
5156	PDGFRA	2.0373	8.6264E-07
419	ART3	-2.1366	9.4508E-07
6039	RNASE6	3.393	1.4395E-06
5660	PSAP	2.2499	1.7211E-06
70	ACTC1	4.144	2.3342E-06
3206	HOXA10	-2.0435	2.7449E-06
7076	TIMP1	3.5344	0.000003021
1192	CLIC1	3.8753	3.1102E-06
710	SERPING1	3.1772	0.000003323
56944	OLFML3	2.2987	0.000003323
10457	GPNMB	3.51	3.3954E-06
341	APOC1	2.2033	3.4579E-06
5328	PLAU	2.4323	3.9242E-06
5532	PPP3CB	-2.4821	4.0896E-06
10234	LRRC17	3.929	5.0968E-06
27190	IL17B	2.2929	5.1213E-06
6945	MLX	-2.71	5.9442E-06
7045	TGFBI	2.6102	5.9734E-06
304	ANXA2P2	3.4362	6.0011E-06
217	ALDH2	-2.4336	6.2925E-06
4016	LOXL1	2.2003	7.4071E-06
23191	CYFIP1	2.5736	8.0083E-06
7572	ZNF24	-2.319	8.3734E-06
4137	MAPT	-2.5669	9.4112E-06
292	SLC25A5	2.9869	9.8404E-06
4621	MYH3	4.8764	0.000010045
2217	FCGRT	2.6275	0.000010063

4681	NBL1	2.1551	0.000010363
55707	NECAP2	2.0162	0.0000122
2275	FHL3	-2.7452	0.000012223
6386	SDCBP	2.6624	0.000012345
6748	SSR4	3.6335	0.000012345
2805	GOT1	-2.2663	0.000012345
3074	HEXB	2.1771	0.000012642
397	ARHGDIB	2.0579	0.000014305
2162	F13A1	2.4647	0.000018004
51375	SNX7	2.4528	0.000018154
2923	PDIA3	2.2143	0.000018773
25937	WWTR1	3.1227	0.000019932
54504	CPVL	2.5661	0.000028686
308	ANXA5	3.4738	0.000029847
10327	AKR1A1	2.22	0.000030207
64747	MFSD1	3.0797	0.00003029
6175	RPLP0	2.592	0.000032154
10095	ARPC1B	2.8596	0.000032401
7188	TRAF5	2.124	0.000033015
10079	ATP9A	2.4104	0.000039406
1339	COX6A2	-2.7042	0.000049366
27242	TNFRSF21	2.644	0.000049969
64231	MS4A6A	3.8055	0.000050739
7048	TGFBR2	3.0782	0.000054357
58526	MID1IP1	-2.2101	0.000055877
3150	HMGN1	3.4279	0.000056744
5359	PLSCR1	2.5122	0.000058082
845	CASQ2	2.6354	0.000060833
58494	JAM2	3.1042	0.000060833
7779	SLC30A1	2.0806	0.0000647
3416	IDE	-2.3998	0.000066367

2542	SLC37A4	-2.0382	0.000068554
7957	EPM2A	-3.4588	0.000070308
214	ALCAM	2.1827	0.000070958
10487	CAP1	2.6769	0.000072765
25813	SAMM50	-2.9978	0.000078795
22919	MAPRE1	2.4596	0.00007902
9056	SLC7A7	2.6294	0.000080897
8655	DYNLL1	3.0503	0.000081779
1634	DCN	3.4286	0.000086752
9332	CD163	3.1751	0.000090462
5479	PPIB	2.7426	0.000090688
5588	PRKCQ	-2.4549	0.000091772
1075	CTSC	2.3756	0.000098459
51642	MRPL48	-2.4435	0.00011171
8826	IQGAP1	2.0718	0.00011238
476	ATP1A1	2.4383	0.00011464
4155	MBP	-2.7208	0.0001202
5641	LGMN	4.2232	0.00015136
11326	VSIG4	2.4684	0.00015721
10840	ALDH1L1	-3.2236	0.00015839
4360	MRC1	3.284	0.00015972
10105	PPIF	-2.1421	0.00016533
6277	S100A6	2.8752	0.00017217
7320	UBE2B	-2.9346	0.00017452
50717	DCAF8	-2.2226	0.00017781
5796	PTPRK	2.1311	0.00018197
55222	LRRC20	-2.5661	0.00018331
5627	PROS1	3.5387	0.00018532
4637	MYL6	2.5251	0.00020287
4330	MN1	-3.9479	0.00021962
10376	TUBA1B	4.5411	0.00022873

293	SLC25A6	2.8711	0.00022906
6122	RPL3	4.176	0.00025146
55365	TMEM176A	2.2511	0.00025542
4013	VWA5A	2.608	0.00025567
81855	SFXN3	2.3685	0.00026175
5270	SERPINE2	2.7434	0.00026465
7846	TUBA1A	4.4543	0.00026943
64581	CLEC7A	2.0502	0.0002842
4046	LSP1	2.7933	0.00030521
8406	SRPX	3.6171	0.00030652
719	C3AR1	2.1411	0.00031089
23139	MAST2	-2.0435	0.00031099
6303	SAT1	3.3773	0.00032392
5223	PGAM1	3.0215	0.00032862
3838	KPNA2	2.3863	0.00037545
960	CD44	4.079	0.00037565
10217	CTDSPL	2.3086	0.00037574
51063	CALHM2	3.5929	0.00038565
473	RERE	-2.1051	0.00038958
3428	IFI16	3.7431	0.00041143
25932	CLIC4	2.6421	0.00041172
54918	CMTM6	2.9047	0.00043675
5355	PLP2	2.3694	0.00047428
11014	KDELR2	2.4609	0.00049117
2200	FBN1	2.5827	0.00049458
10939	AFG3L2	-2.4448	0.00050492
3431	SP110	2.1839	0.000511
60685	ZFAND3	-2.2485	0.00051591
55239	OGFOD1	-2.217	0.00052199
2023	ENO1	2.6531	0.00053624
4282	MIF	4.0647	0.00053624

1915	EEF1A1	4.9139	0.00053867
23358	USP24	-2.0061	0.00054433
8396	PIP4K2B	-2.2593	0.00055025
55208	DCUN1D2	-2.8793	0.00055297
6304	SATB1	-2.0019	0.0005579
3105	HLA-A	2.7895	0.00058137
64761	PARP12	2.0906	0.00059757
57157	PHTF2	-2.4185	0.00060415
5547	PRCP	2.4651	0.00061154
622	BDH1	-2.0577	0.00062495
55577	NAGK	2.6463	0.00063573
4626	MYH8	6.1206	0.00065286
3988	LIPA	2.7859	0.00065346
6324	SCN1B	-4.0275	0.00065389
3177	SLC29A2	-2.5452	0.00066672
5774	PTPN3	-2.8878	0.00068529
718	C3	3.5092	0.00073127
994	CDC25B	2.6087	0.00073943
1397	CRIP2	2.7776	0.00075346
5118	PCOLCE	4.3843	0.00079846
10256	CNKSR1	-2.0712	0.00080474
4060	LUM	4.3237	0.00080683
5757	PTMA	2.4923	0.00081401
6222	RPS18	2.3197	0.0008408
3959	LGALS3BP	3.9413	0.00084419
8483	CILP	3.373	0.0008468
5360	PLTP	3.1142	0.00090108
114876	OSBPL1A	-2.2047	0.00090108
7139	TNNT2	3.3974	0.00091171
7456	WIPF1	3.0692	0.00092058
3490	IGFBP7	2.9953	0.00093531

29109	FHOD1	-2.6169	0.00093531
10486	CAP2	-2.6867	0.0010116
6387	CXCL12	3.9028	0.0010315
3106	HLA-B	2.5766	0.0010528
961	CD47	2.4201	0.0010582
3394	IRF8	2.6877	0.0010791
3587	IL10RA	2.7956	0.0010886
54829	ASPN	4.0681	0.0010886
113146	AHNAK2	2.6128	0.0011081
1292	COL6A2	3.2329	0.0011141
6258	RXRG	-2.698	0.0011303
894	CCND2	2.9859	0.0011422
2532	ACKR1	2.332	0.0011449
108	ADCY2	-2.9495	0.0011554
63935	PCIF1	-2.1473	0.0011554
4218	RAB8A	2.2325	0.0011713
760	CA2	-2.6529	0.0012484
5608	MAP2K6	-2.138	0.0012918
9590	AKAP12	2.0736	0.0012929
567	B2M	2.8175	0.0013138
25878	MXRA5	3.477	0.0013469
10092	ARPC5	3.2685	0.0013557
9909	DENND4B	-2.0838	0.001432
7942	TFEB	-2.2398	0.0014412
2207	FCER1G	2.4557	0.0014427
5728	PTEN	2.061	0.0014606
10418	SPON1	2.1339	0.001484
2212	FCGR2A	2.2393	0.0015018
25816	TNFAIP8	2.8695	0.0015298
11124	FAF1	-3.2751	0.0016223
7422	VEGFA	-2.0426	0.0016801

4067	LYN	2.4569	0.0017165
29108	PYCARD	3.0943	0.0017165
1952	CELSR2	-2.4417	0.0017183
5654	HTRA1	4.0039	0.0017224
1855	DVL1	-2.5856	0.0017251
3275	PRMT2	2.7103	0.0017558
1475	CSTA	2.1659	0.0017558
9168	TMSB10	4.8624	0.0017558
55244	SLC47A1	-2.635	0.0017558
1718	DHCR24	-2.3732	0.0017707
10788	IQGAP2	2.7692	0.0018418
115	ADCY9	-2.5071	0.0018584
10346	TRIM22	2.3447	0.0019099
79630	C1orf54	2.1355	0.0019668
6672	SP100	2.3953	0.0019717
30844	EHD4	2.06	0.0020182
25903	OLFML2B	4.0959	0.0020595
5997	RGS2	2.9318	0.0020725
51296	SLC15A3	2.1152	0.0020725
2059	EPS8	2.39	0.0021297
3075	CFH	2.6224	0.0021444
55803	ADAP2	2.6256	0.0021851
23471	TRAM1	2.6496	0.0021928
5244	ABCB4	-2.8432	0.0022048
9689	BZW1	2.3245	0.0022113
51338	MS4A4A	3.272	0.0022299
6696	SPP1	2.6665	0.0022341
7280	TUBB2A	2.7173	0.0022384
79188	TMEM43	2.2156	0.002289
827	CAPN6	3.533	0.0023598
5179	PENK	2.89	0.0023609

9741	LAPTM4A	2.897	0.0025059
10404	CPQ	2.8336	0.0025357
23585	TMEM50A	2.6564	0.002538
822	CAPG	2.0186	0.0025447
56270	WDR45B	2.2622	0.0025679
5320	PLA2G2A	2.6311	0.002604
9581	PREPL	2.7684	0.002624
23078	VWA8	-2.8562	0.0027432
3840	KPNA4	-2.4801	0.0027833
4209	MEF2D	-2.3028	0.0028261
7412	VCAM1	4.018	0.0028394
1026	CDKN1A	2.3335	0.0028474
5025	P2RX4	2.0893	0.0028952
9111	NMI	2.5118	0.0029018
6664	SOX11	2.1407	0.0029315
6383	SDC2	2.2067	0.0029345
9246	UBE2L6	2.105	0.003047
10723	SLC12A7	2.5851	0.0030868
2335	FN1	2.1226	0.0031101
23129	PLXND1	2.2007	0.0031859
1786	DNMT1	2.0901	0.0032067
11010	GLIPR1	2.0208	0.0032225
2773	GNAI3	2.5202	0.0032418
1293	COL6A3	5.1452	0.0032466
55615	PRR5	2.719	0.003279
81035	COLEC12	3.9143	0.0033259
1352	COX10	-2.6526	0.003337
4636	MYL5	4.0223	0.0033953
3176	HNMT	3.3898	0.0034315
4869	NPM1	2.7684	0.0035569
10777	ARPP21	2.0764	0.0035655

79589	RNF128	-2.5892	0.0035674
3133	HLA-E	2.4209	0.0036304
5445	PON2	2.7415	0.0036327
57017	COQ9	-2.3285	0.0037105
2012	EMP1	2.1149	0.0038588
9817	KEAP1	-2.3713	0.0040622
8519	IFITM1	2.3102	0.0041518
55790	CSGALNACT1	2.2967	0.0042023
4313	MMP2	3.3968	0.0042548
7070	THY1	3.1268	0.0042548
80896	NPL	2.1214	0.0042548
2872	MKNK2	-2.8321	0.0042562
64092	SAMSN1	2.2654	0.0043486
55827	DCAF6	-2.4933	0.0043621
9500	MAGED1	2.003	0.004419
10384	BTN3A3	2.2283	0.0045682
2766	GMPR	-2.6874	0.0047129
51204	TACO1	-2.0416	0.0047371
3839	KPNA3	-2.6457	0.0047473
10051	SMC4	2.2712	0.0047603
5827	PXMP2	-2.2643	0.0048975
7107	GPR137B	3.7129	0.0049259
8878	SQSTM1	-2.7128	0.0049433
8604	SLC25A12	-2.6297	0.0049863
3426	CFI	3.2806	0.0049903
6097	RORC	-2.3527	0.0049999
2124	EVI2B	2.2641	0.0050227
23277	CLUH	-2.3843	0.0050963
3796	KIF2A	2.1441	0.0051851
30008	EFEMP2	2.0656	0.005218
1520	CTSS	3.0459	0.0052492

4853	NOTCH2	3.5845	0.0052796
3109	HLA-DMB	2.3699	0.0052796
27075	TSPAN13	2.6237	0.0052796
51668	HSPB11	2.2453	0.0053049
2192	FBLN1	2.0268	0.0053432
4121	MAN1A1	3.0197	0.0053667
4239	MFAP4	2.1746	0.0053887
3684	ITGAM	2.558	0.0054207
3671	ISLR	2.5491	0.0054477
1266	CNN3	2.6107	0.0054803
7991	TUSC3	2.7406	0.0054803
3927	LASP1	3.1551	0.0054917
1134	CHRNA1	4.2218	0.0056872
1399	CRKL	-2.1813	0.0057998
8897	MTMR3	-2.0542	0.0058182
5157	PDGFRL	2.4825	0.0061152
2123	EVI2A	2.3165	0.0063442
1290	COL5A2	5.3795	0.0063571
5506	PPP1R3A	-2.0413	0.0063831
4047	LSS	-2.8687	0.0064057
26240	FAM50B	-3.0986	0.0064858
10875	FGL2	3.2132	0.0066696
7837	PXDN	2.5818	0.0067371
9201	DCLK1	3.7454	0.0067452
63891	RNF123	-3.2801	0.0067578
51673	TPPP3	2.2846	0.0070627
5533	PPP3CC	-3.256	0.0072688
10094	ARPC3	2.7821	0.0074239
6256	RXRA	-4.2753	0.0074584
2213	FCGR2B	2.0216	0.0075397
9871	SEC24D	2.6554	0.0075589

1601	DAB2	3.4824	0.0078367
1277	COL1A1	4.9413	0.0078909
5334	PLCL1	-2.2181	0.0078951
57088	PLSCR4	2.323	0.0079157
390	RND3	2.2294	0.0079539
5042	PABPC3	3.1528	0.0079643
9540	TP53I3	3.7509	0.0079905
55852	TEX2	-2.4689	0.0079925
7058	THBS2	3.0278	0.0080588
2000	ELF4	2.1393	0.0081283
10462	CLEC10A	2.2505	0.0082486
9061	PAPSS1	2.5205	0.008306
5634	PRPS2	2.2481	0.008319
829	CAPZA1	2.8498	0.008319
1278	COL1A2	5.3137	0.008632
1471	CST3	2.8398	0.0088112
8533	COPS3	-2.8366	0.0088112
2782	GNB1	2.1133	0.0089069
23564	DDAH2	3.178	0.0089519
2191	FAP	2.712	0.0089819
10961	ERP29	2.2029	0.0091686
7867	MAPKAPK3	-2.022	0.0094857
8531	YBX3	-2.2832	0.0096056
23075	SWAP70	2.1091	0.0098155
56829	ZC3HAV1	2.603	0.0098937
5720	PSME1	2.5699	0.0099957
4692	NDN	2.9208	0.010038
11275	KLHL2	2.2978	0.010107
51809	GALNT7	2.9057	0.010163
58533	SNX6	2.4264	0.010355
6640	SNTA1	-3.0982	0.010381

63941	NECAB3	-2.4137	0.010518
3052	HCCS	-2.2004	0.010551
1176	AP3S1	2.875	0.010668
58513	EPS15L1	-2.3432	0.010799
65108	MARCKSL1	2.0352	0.010809
4232	MEST	3.8807	0.010885
22856	CHSY1	2.9596	0.011014
5788	PTPRC	2.518	0.011019
6218	RPS17	2.0762	0.011032
81563	C1orf21	-2.4439	0.011048
6777	STAT5B	-2.3887	0.011052
79001	VKORC1	2.4045	0.011198
29760	BLNK	2.6675	0.011427
596	BCL2	-2.3317	0.011427
25822	DNAJB5	-2.4993	0.01165
5436	POLR2G	2.1384	0.011692
2534	FYN	3.4316	0.011717
3091	HIF1A	3.4286	0.011953
55168	MRPS18A	-2.117	0.012076
5325	PLAGL1	3.4584	0.012253
23654	PLXNB2	3.0656	0.012335
55144	LRRC8D	2.4138	0.012466
22795	NID2	3.3565	0.012481
3059	HCLS1	3.1818	0.012574
3134	HLA-F	2.3437	0.012674
3107	HLA-C	2.9617	0.012674
91977	MYOZ3	-2.1098	0.012833
834	CASP1	2.9515	0.012925
32	ACACB	-2.4399	0.01308
7077	TIMP2	3.0618	0.013087
64208	POPDC3	-2.5889	0.013093

10549	PRDX4	2.7278	0.01315
6890	TAP1	2.132	0.01315
10049	DNAJB6	-2.2446	0.01315
2874	GPS2	-2.7111	0.01315
29780	PARVB	-2.6452	0.013185
5791	PTPRE	2.8592	0.013304
8266	UBL4A	-2.1644	0.013858
10749	KIF1C	-2.0587	0.013978
2824	GPM6B	2.5762	0.014111
678	ZFP36L2	2.0656	0.014138
1650	DDOST	2.0793	0.014434
23194	FBXL7	2.44	0.014543
51306	FAM13B	2.4874	0.014609
54977	SLC25A38	-2.1257	0.014819
6717	SRI	2.7511	0.014864
7373	COL14A1	2.5478	0.014952
23423	TMED3	3.2135	0.014972
10161	LPAR6	2.5525	0.015028
3249	HPN	-2.8065	0.015107
1808	DPYSL2	3.3249	0.015107
6135	RPL11	2.314	0.015221
11034	DSTN	2.2281	0.015221
199	AIF1	2.5587	0.015221
2171	FABP5	2.379	0.015297
694	BTG1	2.7666	0.015332
2719	GPC3	2.3184	0.015451
9670	IPO13	-2.3988	0.015699
55324	ABCF3	-2.4775	0.015785
55544	RBM38	-2.1264	0.016303
11075	STMN2	2.0437	0.016372
79895	ATP8B4	2.1556	0.016417

51	ACOX1	-2.1761	0.016575
6451	SH3BGRL	3.766	0.016577
5480	PPIC	2.3715	0.016603
114908	TMEM123	2.8478	0.016768
10920	COPS8	-2.4404	0.017027
1902	LPAR1	2.5315	0.01726
4734	NEDD4	-2.0255	0.01726
7074	TIAM1	2.5227	0.017433
81031	SLC2A10	3.3148	0.017518
3956	LGALS1	2.5031	0.017617
23136	EPB41L3	4.5488	0.017648
1842	ECM2	2.185	0.017698
10159	ATP6AP2	3.1971	0.017777
23034	SAMD4A	-3.9955	0.017873
7803	PTP4A1	-2.2855	0.017987
9445	ITM2B	2.845	0.018017
4088	SMAD3	-2.2511	0.018151
57061	HYMAI	2.5476	0.018231
79971	WLS	2.2881	0.018232
55619	DOCK10	2.1723	0.018485
25820	ARIH1	-2.6703	0.018539
3087	HHEX	2.027	0.018659
1281	COL3A1	6.5666	0.018725
29803	REPIN1	-2.1089	0.018725
2697	GJA1	2.521	0.018794
143	PARP4	3.4142	0.018959
79894	ZNF672	-2.5838	0.018959
23598	PATZ1	-2.2894	0.019135
6575	SLC20A2	-2.1235	0.019148
115207	KCTD12	3.5878	0.019148
27183	VPS4A	-2.5896	0.019156

6678	SPARC	6.0422	0.019283
5567	PRKACB	2.947	0.019568
10572	SIVA1	-2.4475	0.019568
3915	LAMC1	2.7388	0.019722
2326	FMO1	3.6579	0.019955
113	ADCY7	3.555	0.019964
22887	FOXJ3	-2.5485	0.020028
5937	RBMS1	2.3964	0.020157
91851	CHRDL1	2.9827	0.020186
23303	KIF13B	2.3889	0.020323
5621	PRNP	2.3574	0.0205
901	CCNG2	2.6572	0.020587
196	AHR	3.4963	0.020594
9640	ZNF592	-2.5209	0.020594
90865	IL33	2.6148	0.020594
5696	PSMB8	2.7855	0.020625
54477	PLEKHA5	2.4285	0.021678
2314	FLII	-3.1826	0.022136
120	ADD3	2.4724	0.022201
1805	DPT	3.4611	0.022409
22884	WDR37	-2.3896	0.022457
1193	CLIC2	2.0085	0.022836
90410	IFT20	2.9062	0.02285
25825	BACE2	2.0406	0.023031
6091	ROBO1	3.1271	0.023271
389136	VGLL3	2.8179	0.023794
26099	SZRD1	-2.0745	0.023885
79161	TMEM243	2.3768	0.023927
4969	OGN	3.183	0.023958
23125	CAMTA2	-2.1269	0.024078
963	CD53	2.3913	0.024156

378	ARF4	2.5627	0.024224
4082	MARCKS	3.5828	0.024297
4286	MITF	-2.7343	0.024495
6535	SLC6A8	-2.2909	0.025268
7089	TLE2	-3.0697	0.025404
6390	SDHB	-2.0897	0.025447
54583	EGLN1	-2.6569	0.025652
5836	PYGL	2.747	0.025713
27230	SERP1	2.6294	0.025795
9513	FXR2	-2.6633	0.025878
1647	GADD45A	3.2174	0.025978
4928	NUP98	-2.1553	0.026142
7059	THBS3	2.4844	0.026353
1282	COL4A1	2.2447	0.026521
1289	COL5A1	5.1677	0.026688
1519	CTSO	2.1984	0.026777
5908	RAP1B	2.8308	0.026818
28996	HIPK2	-2.2825	0.026827
5833	PCYT2	-2.14	0.026933
55608	ANKRD10	2.3501	0.026933
57380	MRS2	-2.1335	0.027004
8910	SGCE	2.8986	0.027083
6746	SSR2	2.836	0.027257
55076	TMEM45A	2.8312	0.027347
1513	CTSK	3.7257	0.027624
7764	ZNF217	3.1986	0.027826
23062	GGA2	-2.0846	0.027831
6745	SSR1	3.6159	0.028104
9839	ZEB2	3.2294	0.028104
4004	LMO1	-2.0087	0.028104
11177	BAZ1A	2.0238	0.028104

7106	TSPAN4	2.6345	0.028241
5168	ENPP2	2.7326	0.028469
2764	GMFB	2.2434	0.028495
9780	PIEZO1	2.6139	0.028664
79784	MYH14	-2.942	0.028664
57415	C3orf14	2.1836	0.028664
1612	DAPK1	2.285	0.028711
491	ATP2B2	-2.7131	0.029271
11155	LDB3	-2.186	0.029381
51280	GOLM1	2.6309	0.029916
5536	PPP5C	-2.0383	0.030135
8165	AKAP1	-2.0127	0.030192
2195	FAT1	3.6334	0.030198
51279	C1RL	2.3362	0.03028
283298	OLFML1	2.6368	0.030337
10178	TENM1	2.8699	0.030417
10950	BTG3	2.7759	0.030763
2173	FABP7	-2.024	0.030837
23179	RGL1	2.4294	0.030896
3479	IGF1	2.1137	0.03093
2288	FKBP4	-2.7777	0.031447
55905	RNF114	-2.3379	0.031922
51196	PLCE1	2.0072	0.031922
2029	ENSA	-2.0736	0.032178
6899	TBX1	-2.6115	0.032197
1845	DUSP3	-2.6056	0.032397
1580	CYP4B1	-2.3667	0.032497
91754	NEK9	-2.2596	0.032639
1495	CTNNA1	2.1812	0.03283
4122	MAN2A2	-3.1299	0.03283
2065	ERBB3	3.1583	0.032861

327	APEH	-2.2282	0.032911
6934	TCF7L2	2.657	0.032911
55665	URGCP	-2.0188	0.032911
2037	EPB41L2	2.0067	0.03306
11047	ADRM1	-2.4425	0.033146
2992	GYG1	-2.6982	0.03324
7306	TYRP1	-3.827	0.033266
6201	RPS7	2.8438	0.033706
56288	PARD3	-2.0209	0.033745
55778	ZNF839	-2.3357	0.03378
11015	KDELR3	2.2118	0.03402
9261	MAPKAPK2	-2.1385	0.03412
23522	KAT6B	-2.0161	0.034349
22877	MLXIP	-3.6795	0.034611
7866	IFRD2	-2.1945	0.034611
5442	POLRMT	-2.7653	0.035043
10769	PLK2	3.1052	0.035072
2004	ELK3	2.8837	0.035227
56271	BEX4	3.1834	0.035785
8228	PNPLA4	-2.4546	0.035844
9321	TRIP11	-2.0463	0.035844
23150	FRMD4B	2.6165	0.035872
51207	DUSP13	-2.1004	0.035923
8694	DGAT1	-2.0423	0.036048
55652	SLC48A1	-3.1676	0.036085
83468	GLT8D2	2.7507	0.03617
65018	PINK1	-2.1375	0.036586
9825	SPATA2	-2.4678	0.036752
26064	RAI14	2.8019	0.036975
8560	DEGS1	2.4099	0.037452
9770	RASSF2	3.1159	0.037909

6133	RPL9	2.6798	0.038122
9902	MRC2	2.2785	0.038246
5178	PEG3	2.427	0.038262
440738	MAP1LC3C	2.0164	0.038392
10603	SH2B2	-2.4179	0.038575
11066	SNRNP35	-2.1109	0.038939
3297	HSF1	-2.3178	0.039351
55830	GLT8D1	2.7192	0.039446
4842	NOS1	-2.1529	0.039463
51339	DACT1	2.0473	0.039496
857	CAV1	2.3776	0.039632
79805	VASH2	2.4494	0.039632
51728	POLR3K	-2.0647	0.039646
114327	EFHC1	2.8296	0.039745
10659	CELF2	3.5995	0.039802
81558	FAM117A	2.0179	0.039844
3778	KCNMA1	-2.0581	0.039934
2205	FCER1A	2.2816	0.04053
55662	HIF1AN	-2.1985	0.040591
10444	ZER1	-2.7538	0.040882
2289	FKBP5	-2.2827	0.040923
6944	VPS72	-2.5145	0.040954
4015	LOX	3.0076	0.040985
3069	HDLBP	-2.4614	0.041121
5116	PCNT	-2.1313	0.041382
27129	HSPB7	-2.0946	0.041719
1306	COL15A1	3.6405	0.041809
9217	VAPB	-2.4016	0.042321
1837	DTNA	-2.2148	0.042321
5830	PEX5	-2.1126	0.042367
54453	RIN2	2.2312	0.042963

10857	PGRMC1	2.785	0.043807
5348	FXYD1	-2.0172	0.043936
57162	PELI1	2.6643	0.044759
10456	HAX1	-2.2893	0.044947
2873	GPS1	-2.3732	0.045107
637	BID	2.0762	0.04548
23014	FBXO21	-2.7647	0.046187
51526	OSER1	-2.9929	0.046503
55326	AGPAT5	2.0569	0.046503
50856	CLEC4A	2.0143	0.046513
23023	TMCC1	-2.8478	0.046535
9148	NEURL1	-2.2483	0.046747
10493	VAT1	2.0922	0.047146
145389	SLC38A6	2.0411	0.047288
23270	TSPYL4	2.1227	0.047442
22921	MSRB2	-2.6197	0.04764
5048	PAFAH1B1	-2.1163	0.04788
256987	SERINC5	2.7153	0.048186
64960	MRPS15	-2.1047	0.048394
2101	ESRRA	-2.5375	0.049125
10971	YWHAQ	3.6866	0.049202
3912	LAMB1	3.684	0.049461
23312	DMXL2	2.7138	0.049461
51170	HSD17B11	2.6385	0.049574
28999	KLF15	-2.3231	0.049898

Suppl. Table 2. List of genes belonging to the DMD signature network.

Name	Combined ES	Adj. Pval	Biological Process (GO)
HLA-DPB1	2.8816	0	GO:0032729 positive regulation of interferon-gamma production;GO:0060333 interferon-gamma-mediated signaling pathway;GO:0019886 antigen processing and presentation of exogenous peptide antigen via MHC class II
VAMP8	2.9953	0	GO:1903595 positive regulation of histamine secretion by mast cell;GO:0016240 autophagosome membrane docking;GO:0002278 eosinophil activation involved in immune response
MAGED2	3.0779	0	GO:0070294 renal sodium ion absorption;GO:0003096 renal sodium ion transport;GO:0070293 renal absorption
HLA-DPA1	2.9842	0	GO:0032729 positive regulation of interferon-gamma production;GO:0060333 interferon-gamma-mediated signaling pathway;GO:0019886 antigen processing and presentation of exogenous peptide antigen via MHC class II
FTL	3.3021	0	GO:0006880 intracellular sequestering of iron ion;GO:0097577 sequestering of iron ion;GO:0051238 sequestering of metal ion
A2M	2.4783	2.06E-13	GO:0001868 regulation of complement activation, lectin pathway;GO:0001869 negative regulation of complement activation, lectin pathway;GO:0045916 negative regulation of complement activation
FSTL1	2.3913	7.54E-13	GO:0061484 hematopoietic stem cell homeostasis;GO:0030510 regulation of BMP signaling pathway;GO:0045446 endothelial cell differentiation
ITGB2	2.3645	8.33E-13	GO:0045914 negative regulation of catecholamine metabolic process;GO:0045963 negative regulation of dopamine metabolic process;GO:2000361 regulation of prostaglandin-E synthase activity
ADAM10	2.2241	6.60E-12	GO:0051089 constitutive protein ectodomain proteolysis;GO:0035333 Notch receptor processing, ligand-dependent;GO:0098696 regulation of neurotransmitter receptor localization to postsynaptic specialization membrane
AXL	2.1538	1.92E-11	GO:0097350 neutrophil clearance;GO:0032825 positive regulation of natural killer cell differentiation;GO:0048549 positive regulation of pinocytosis
HLA-DQB1	2.1338	2.77E-11	GO:0002381 immunoglobulin production involved in immunoglobulin mediated immune response;GO:0060333 interferon-gamma-mediated signaling pathway;GO:0019886 antigen processing and presentation of exogenous peptide antigen via MHC class II
SRGN	2.0239	1.16E-10	GO:0033371 T cell secretory granule organization;GO:0033367 protein localization to mast cell secretory granule;GO:0033368 protease localization to mast cell secretory granule
CSF1R	3.5282	1.06E-09	GO:0038145 macrophage colony-stimulating factor signaling pathway;GO:0120041 positive regulation of macrophage proliferation;GO:0120040 regulation of macrophage proliferation
CD74	3.1564	5.66E-09	GO:2000448 positive regulation of macrophage migration inhibitory factor signaling pathway;GO:0002901 mature B cell apoptotic process;GO:0002905 regulation of mature B cell apoptotic process
TYROBP	4.0016	2.33E-08	GO:0110090 positive regulation of hippocampal neuron apoptotic process;GO:0002222 stimulatory killer cell immunoglobulin-like receptor signaling pathway;GO:0090634 microglial cell mediated cytotoxicity
HLA-DRA	3.9545	3.13E-08	GO:0002491 antigen processing and presentation of endogenous peptide antigen via MHC class II;GO:0002469 myeloid dendritic cell antigen processing and presentation;GO:0002399 MHC class II protein complex assembly
IGFBP4	2.0655	4.41E-08	GO:0043568 positive regulation of insulin-like growth factor receptor signaling pathway;GO:0044342 type B pancreatic cell proliferation;GO:0043567 regulation of insulin-like growth factor receptor signaling pathway
TRIP10	-2.8064	1.31E-07	GO:0051056 regulation of small GTPase mediated signal transduction;GO:0007264 small GTPase mediated signal transduction;GO:0030036 actin cytoskeleton organization
MXRA8	2.2948	4.82E-07	GO:0060857 establishment of glial blood-brain barrier;GO:0060856 establishment of blood-brain barrier;GO:0021782 glial cell development
SERPING1	3.1772	3.32E-06	GO:0001868 regulation of complement activation, lectin pathway;GO:0001869 negative regulation of complement activation, lectin pathway;GO:0045916 negative regulation of complement activation
CYFIP1	2.5736	8.01E-06	GO:0099578 regulation of translation at postsynapse, modulating synaptic transmission;GO:0140245 regulation of translation at postsynapse;GO:1903422 negative regulation of synaptic vesicle recycling
NECAP2	2.0162	1.22E-05	GO:0006897 endocytosis;GO:0098657 import into cell;GO:0015031 protein transport
F13A1	2.4647	1.80E-05	GO:0072378 blood coagulation, fibrin clot formation;GO:0018149 peptide cross-linking;GO:0002576 platelet degranulation
ANXA5	3.4738	2.98E-05	GO:0050819 negative regulation of coagulation;GO:0050818 regulation of coagulation;GO:0002576 platelet degranulation
ARPC1B	2.8596	3.24E-05	GO:0034314 Arp2/3 complex-mediated actin nucleation;GO:0045010 actin nucleation;GO:0043627 response to estrogen
IQGAP1	2.0718	0.000112	GO:1900086 positive regulation of peptidyl-tyrosine autophosphorylation;GO:1900084 regulation of peptidyl-tyrosine autophosphorylation;GO:0072015 glomerular visceral epithelial cell development
PROS1	3.5387	0.00018	GO:0042730 fibrinolysis;GO:0030195 negative regulation of blood coagulation;GO:1900047 negative

		5	regulation of hemostasis
CD44	4.0790	0.000376	GO:1900623 regulation of monocyte aggregation;GO:1900625 positive regulation of monocyte aggregation;GO:0070487 monocyte aggregation
FBN1	2.5827	0.000495	GO:0035582 sequestering of BMP in extracellular matrix;GO:0035583 sequestering of TGFbeta in extracellular matrix;GO:0048050 post-embryonic eye morphogenesis
C3	3.5092	0.000731	GO:0001798 positive regulation of type IIa hypersensitivity;GO:0002894 positive regulation of type II hypersensitivity;GO:0001970 positive regulation of activation of membrane attack complex
CNKSR1	-2.0712	0.000805	GO:0007266 Rho protein signal transduction;GO:0007265 Ras protein signal transduction;GO:0007264 small GTPase mediated signal transduction
WIPF1	3.0692	0.000921	GO:0002433 immune response-regulating cell surface receptor signaling pathway involved in phagocytosis;GO:0038096 Fc-gamma receptor signaling pathway involved in phagocytosis;GO:0038094 Fc-gamma receptor signaling pathway
IGFBP7	2.9953	0.000935	GO:0051414 response to cortisol;GO:0007566 embryo implantation;GO:0050810 regulation of steroid biosynthetic process
CXCL12	3.9028	0.001032	GO:1990478 response to ultrasound;GO:1903237 negative regulation of leukocyte tethering or rolling;GO:0038146 chemokine (C-X-C motif) ligand 12 signaling pathway
IL10RA	2.7956	0.001089	GO:0070086 ubiquitin-dependent endocytosis;GO:0010507 negative regulation of autophagy;GO:0046427 positive regulation of JAK-STAT cascade
ARPC5	3.2685	0.001356	GO:0021769 orbitofrontal cortex development;GO:0051639 actin filament network formation;GO:0030011 maintenance of cell polarity
FCER1G	2.4557	0.001443	GO:0038156 interleukin-3-mediated signaling pathway;GO:0071404 cellular response to low-density lipoprotein particle stimulus;GO:0032753 positive regulation of interleukin-4 production
PTEN	2.0610	0.001461	GO:2000808 negative regulation of synaptic vesicle clustering;GO:0051548 negative regulation of keratinocyte migration;GO:1903984 positive regulation of TRAIL-activated apoptotic signaling pathway
FCGR2A	2.2393	0.001502	GO:0002433 immune response-regulating cell surface receptor signaling pathway involved in phagocytosis;GO:0038096 Fc-gamma receptor signaling pathway involved in phagocytosis;GO:0038094 Fc-gamma receptor signaling pathway
LYN	2.4569	0.001717	GO:0070668 positive regulation of mast cell proliferation;GO:0070667 negative regulation of mast cell proliferation;GO:0070447 positive regulation of oligodendrocyte progenitor proliferation
PENK	2.89	0.002361	GO:0051867 general adaptation syndrome, behavioral process;GO:0099538 synaptic signaling via neuropeptide;GO:0051866 general adaptation syndrome
SDC2	2.2067	0.002935	GO:0006027 glycosaminoglycan catabolic process;GO:0006026 aminoglycan catabolic process;GO:0048013 ephrin receptor signaling pathway
GNAI3	2.5202	0.003242	GO:0033864 positive regulation of NAD(P)H oxidase activity;GO:0033860 regulation of NAD(P)H oxidase activity;GO:0032930 positive regulation of superoxide anion generation
ITGAM	2.5580	0.005421	GO:0045914 negative regulation of catecholamine metabolic process;GO:0045963 negative regulation of dopamine metabolic process;GO:0110090 positive regulation of hippocampal neuron apoptotic process
ISLR	2.5491	0.005448	GO:0002576 platelet degranulation;GO:0045055 regulated exocytosis;GO:0006887 exocytosis
CHRNA1	4.2218	0.005687	GO:0048630 skeletal muscle tissue growth;GO:0046716 muscle cell cellular homeostasis;GO:0007274 neuromuscular synaptic transmission
CRKL	-2.1813	0.0058	GO:0035685 helper T cell diapedesis;GO:0035684 helper T cell extravasation;GO:0098749 cerebellar neuron development
ARPC3	2.7821	0.007424	GO:0070358 actin polymerization-dependent cell motility;GO:0034314 Arp2/3 complex-mediated actin nucleation;GO:1990090 cellular response to nerve growth factor stimulus
FCGR2B	2.0216	0.00754	GO:0001811 negative regulation of type I hypersensitivity;GO:0001797 negative regulation of type IIa hypersensitivity;GO:0001814 negative regulation of antibody-dependent cellular cytotoxicity
DAB2	3.4824	0.007837	GO:0035026 leading edge cell differentiation;GO:2000370 positive regulation of clathrin-dependent endocytosis;GO:2000643 positive regulation of early endosome to late endosome transport
PAPSS1	2.5205	0.008306	GO:0000103 sulfate assimilation;GO:0034036 purine ribonucleoside bisphosphate biosynthetic process;GO:0050428 3'-phosphoadenosine 5'-phosphosulfate biosynthetic process
CST3	2.8398	0.008811	GO:0010711 negative regulation of collagen catabolic process;GO:0060311 negative regulation of elastin catabolic process;GO:0060313 negative regulation of blood vessel remodeling
ZC3HAV1	2.6030	0.009894	GO:0070212 protein poly-ADP-ribosylation;GO:0006471 protein ADP-ribosylation;GO:0061014 positive regulation of mRNA catabolic process
AP3S1	2.8750	0.010668	GO:0048490 anterograde synaptic vesicle transport;GO:0099514 synaptic vesicle cytoskeletal transport;GO:0099517 synaptic vesicle transport along microtubule
EPS15L1	-2.3432	0.010799	GO:0042059 negative regulation of epidermal growth factor receptor signaling pathway;GO:1901185 negative regulation of ERBB signaling pathway;GO:0042058 regulation of epidermal growth factor receptor signaling pathway
PTPRC	2.5180	0.011019	GO:1904155 DN2 thymocyte differentiation;GO:0044855 plasma membrane raft distribution;GO:0044856 plasma membrane raft localization
STAT5B	-2.3887	0.01105	GO:0046544 development of secondary male sexual characteristics;GO:0046543 development of

		2	secondary female sexual characteristics;GO:0032825 positive regulation of natural killer cell differentiation
BLNK	2.6675	0.011427	GO:0050853 B cell receptor signaling pathway;GO:0030183 B cell differentiation;GO:0050851 antigen receptor-mediated signaling pathway
FYN	3.4316	0.011717	GO:0000304 response to singlet oxygen;GO:1905430 cellular response to glycine;GO:1905429 response to glycine
GPC3	2.3184	0.015451	GO:0072138 mesenchymal cell proliferation involved in ureteric bud development;GO:0072180 mesonephric duct morphogenesis;GO:0061209 cell proliferation involved in mesonephros development
TIAM1	2.5227	0.017433	GO:1904266 regulation of Schwann cell chemotaxis;GO:1904268 positive regulation of Schwann cell chemotaxis;GO:1990751 Schwann cell chemotaxis
SPARC	6.0422	0.,019283	GO:0033591 response to L-ascorbic acid;GO:0009629 response to gravity;GO:0010288 response to lead ion
PRKACB	2.9470	0.019568	GO:0097338 response to clozapine;GO:0097332 response to antipsychotic drug;GO:1901621 negative regulation of smoothened signaling pathway involved in dorsal/ventral neural tube patterning
LAMC1	2.7388	0.019722	GO:0031581 hemidesmosome assembly;GO:0035633 maintenance of permeability of blood-brain barrier;GO:0007492 endoderm development
CHRDL1	2.9827	0.020186	GO:0030514 negative regulation of BMP signaling pathway;GO:0030510 regulation of BMP signaling pathway;GO:0090101 negative regulation of transmembrane receptor protein serine/threonine kinase signaling pathway
RAP1B	2.8308	0.026818	GO:2000301 negative regulation of synaptic vesicle exocytosis;GO:1902804 negative regulation of synaptic vesicle transport;GO:0097211 cellular response to gonadotropin-releasing hormone
GOLM1	2.6309	0.029916	GO:0006997 nucleus organization;GO:0043687 post-translational protein modification;GO:0019216 regulation of lipid metabolic process
CTNNA1	2.1812	0.03283	GO:2001045 negative regulation of integrin-mediated signaling pathway;GO:0071680 response to indole-3-methanol;GO:0071681 cellular response to indole-3-methanol
ERBB3	3.1583	0.032861	GO:0070886 positive regulation of calcineurin-NFAT signaling cascade;GO:0106058 positive regulation of calcineurin-mediated signaling;GO:0070884 regulation of calcineurin-NFAT signaling cascade
CAV1	2.3776	0.039632	GO:1900085 negative regulation of peptidyl-tyrosine autophosphorylation;GO:0038016 insulin receptor internalization;GO:0033484 nitric oxide homeostasis
FCER1A	2.2816	0.04053	GO:0038095 Fc-epsilon receptor signaling pathway;GO:0038093 Fc receptor signaling pathway;GO:0002768 immune response-regulating cell surface receptor signaling pathway
LAMB1	3.6840	0.049461	GO:0021812 neuronal-glial interaction involved in cerebral cortex radial glia guided migration;GO:0070831 basement membrane assembly;GO:0021801 cerebral cortex radial glia guided migration
SPP1	2.6665	0.002234	GO:0048673 collateral sprouting of intact axon in response to injury;GO:0048683 regulation of collateral sprouting of intact axon in response to injury;GO:0048685 negative regulation of collateral sprouting of intact axon in response to injury
IGF1	2.1137	0.03093	GO:1904073 regulation of trophectodermal cell proliferation;GO:1904075 positive regulation of trophectodermal cell proliferation;GO:0009441 glycolate metabolic process
FN1	2.1226	0.00311	GO:1904235 regulation of substrate-dependent cell migration, cell attachment to substrate;GO:1904237 positive regulation of substrate-dependent cell migration, cell attachment to substrate;GO:0006931 substrate-dependent cell migration, cell attachment to substrate
TIMP1	3.5344	0.000003	GO:0002248 connective tissue replacement involved in inflammatory response wound healing;GO:0002246 wound healing involved in inflammatory response;GO:1901164 negative regulation of trophoblast cell migration
MMP2	3.3968	0.004255	GO:0001957 intramembranous ossification;GO:0036072 direct ossification;GO:0001955 blood vessel maturation
